# Methods for Involving Older People in Health Research—A Review of the Literature

**DOI:** 10.3390/ijerph14121476

**Published:** 2017-11-29

**Authors:** Imke Schilling, Ansgar Gerhardus

**Affiliations:** 1 Department for Health Services Research, Institute of Public Health and Nursing Research, University of Bremen, Grazer Straße 4, 28359 Bremen, Germany; ansgar.gerhardus@uni-bremen.de; 2 Health Sciences Bremen, University of Bremen, 28359 Bremen, Germany

**Keywords:** PPI, patient and public involvement, older people, people with old-age-related conditions, diversity, health research, review

## Abstract

Demographic change has increased the need for research on healthcare for older people. Recently there has been a growing awareness that research might benefit from actively involving patients and the public in study design and conduct. Besides empowering patients and democratizing research, involvement enhances the quality of research and the development of equitable healthcare solutions. Little is known about how to involve older people. This review aims to support scientists intending to involve older people in health research by systematically identifying and describing studies involving older people and analyzing associated facilitators and challenges. Old people were operationalized as people living with old-age-related conditions. We conducted a systematic search in PubMed, CINAHL (Cumulative Index to Nursing and Allied Health Literature), and Cochrane library for the period 2007 to July 2017 and also manually searched reference lists of the nine retrieved articles and other relevant sources. While involvement of older people in research is feasible, specific challenges related to this group need be taken into account. Strategies to enhance effective involvement comprise a thoughtful choice of location, use of visualization and accessible communication, building good relationships and flexible approaches. Further research is needed on the involvement of people in care homes or with vision, hearing or mobility limitations.

## 1. Introduction

Due to demographic change, healthcare for older people is gaining more importance. Consequently, the need for health research that focuses on older people is increasing. In the last years there has been a growing awareness that patients and the public should be more actively involved in the design, conduct and dissemination of health-related studies [1,2]. It has been argued that patient and public involvement (PPI) leads to a democratization of the research process and supports the empowerment of patients, especially of the easily overlooked [1,2,3,4,5,6]. PPI enhances the development of equitable healthcare solutions, changes health outcomes, and is thus a prerequisite for a patient-centered health care [2,7,8].

While there is still no unified definition of PPI, INVOLVE, the national advisory group to the National Health System (NHS) in the United Kingdom (UK), describes involvement as “research being carried out ‘with’ or ‘by’ members of the public rather than ‘to’, ‘about’ or ‘for’ them” [9]. The Patient-Centered Research Outcomes Institute (PCORI) in the United States (US) and the Canadian Institutes of Health Research (CIHR) use the terms ‘engagement in research’ [10] or ‘patient engagement’ [7]. Similarly to INVOLVE, these two organizations define these terms as the active and meaningful involvement of patients or other stakeholders in research. For the sake of consistency we used the terms and the definition of PPI from INVOLVE throughout our review.

Often, PPI is described as a continuum, from low to high degree of participation [4,11,12,13]. Mostly, three levels of involvement are distinguished: The lowest level is ‘consultation’, meaning that research will ask patients and/or the public for their views. ‘Collaboration’ is a partnership between researchers and the public/patients that strives for continuity; the public/patients take part in decisions. The highest level is ‘user-led’, where the public/patients are supported to lead parts of the research themselves. Most recently in the UK there has been a shift from the categories of consultation, collaboration and user-led to co-production approaches [14]. While an agreed definition of co-production is still missing, central principles such as reciprocity and mutuality were introduced by Boyle et al. [15].

Patients and/or the public can be involved in all or single stages of the research process. Bagley et al. [16] specify a pathway for PPI in clinical trials that starts with defining the most relevant research questions and ends with measuring the impact of the findings and informing future trials. In a systematic review, Domecq et al. [1] found that the most common methods of patient engagement were focus groups, interviews, and surveys. Not all of these methods of engagement would, however, pass INVOLVE’s definition of involvement. According to the review, more active participation was achieved by memberships in study boards or advisory councils, or through attending regular meetings with researchers. Further, in some studies participants got involved in operational tasks, such as recruitment procedures, data collection, or measuring outcomes [1]. The authors noted that most studies use convenience samples for recruiting and selecting patients, and only very few select a random sample of participants [1]. Self-selection of participants might lead to limited diversity of the group and decrease equality of opportunity to get involved in research. Among others, people with old-age-related conditions are ‘easily overlooked’ (a term preferred by INVOLVE to ‘hard-to-reach’, as the latter suggests that the fault is on the site of the patients) or excluded due to stigmata that devaluate their abilities to contribute [17,18,19]. Being underrepresented in PPI can lead to the perspectives of patients/the public not being heard in research activities and in the resulting healthcare [17,18,20]. Based on case studies, Iliffe et al. [21] see challenges in the involvement of people with progressive disease, cognitive impairments and limitations in mobility or speech, and suggest that disease specific issues should be considered in the planning of PPI. However, while there has been an increasing body of research on involving patients in general, there is no overview on studies that actively involved people with old-age-related conditions.

The aim of this review is to support scientists who intend to involve older patients in health research by systematically identifying and describing studies that involved older patients and analyzing the associated facilitators and challenges. We used the term ‘patient’ throughout the article for consistency, but are aware that other terms such as ‘service users’ would also fit and may better display the active role that PPI strives for. For the purpose of this study health research is understood as bio-medical basic research, clinical research, medical (bio-)technology research, pharmaceutical research, health services research and public health research. To operationalize the term older people, we deliberately did not define a certain age group but conditions that are associated with old age. We found a range of conditions that are highly relevant for older people [21,22] based on which we focused on six conditions that can possibly induce additional challenges for PPI: people with dementia, frailty, people in need of care who live in a nursing home, people with reduced mobility, as well as people with advanced hearing or visual impairments.

Our research questions were: (a) What methods of PPI have been used to involve people with six relevant old-age-related conditions in health research? (b) Which facilitators and challenges of PPI have been reported in these studies?

## 2. Methods

### 2.1. Eligibility Criteria

We searched for methods to actively involve people with old-age-related conditions in health research. Articles had to fit to INVOLVE’s definition of PPI in research which states, “research being carried out ‘with’ or ‘by’ members of the public rather than ‘to’, ‘about’ or ‘for’ them” [9]. We only included peer-reviewed empirical articles. Reviews, editorials, opinions and comments were excluded but used to inform the background, analysis and discussion. Table 1 lists all eligibility criteria in detail.

### 2.2. Search Strategy

**Figure 1:**
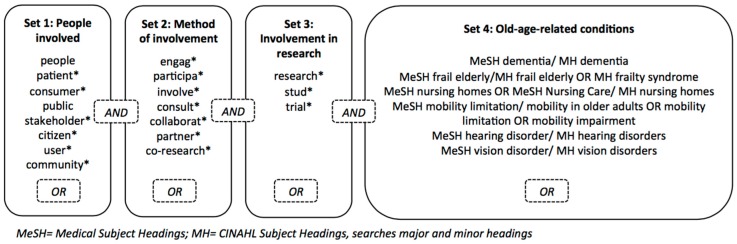
Search Terms.

A systematic search in three electronic databases comprising medical or nursing literature (PubMed, CINAHL (Cumulative Index to Nursing and Allied Health Literature) and Cochrane library) was undertaken from 1 January 2007 to 21 July 2017 following the Preferred Reporting Items for Systematic Reviews and Meta-Analyses (PRISMA) Guidelines [23]. Our search combined sets of terms (see Figure 1) for the people involved (set 1), the method of involvement (set 2), the involvement in research (set 3) and the six old-age-related conditions (set 4). To improve accuracy we followed Boote et al. [3] and limited the word stem ‘participa*’ in set 2 to articles that also have ‘involv*’ or ‘research’ in their title or abstract. In set 4 the terms in front of the slash were used in PubMed and Cochrane library. The terms behind the slash were used in CINAHL.

In addition, manual searching of reference lists of pertinent articles, of the specialist journal ‘Research involvement and engagement’, and the INVOLVE library ‘Putting it into practice’ was conducted. We further used citation tracking in Google Scholar to identify articles that cited included articles and screened them for eligibility.

### 2.3. Article Selection

**Figure 2:**
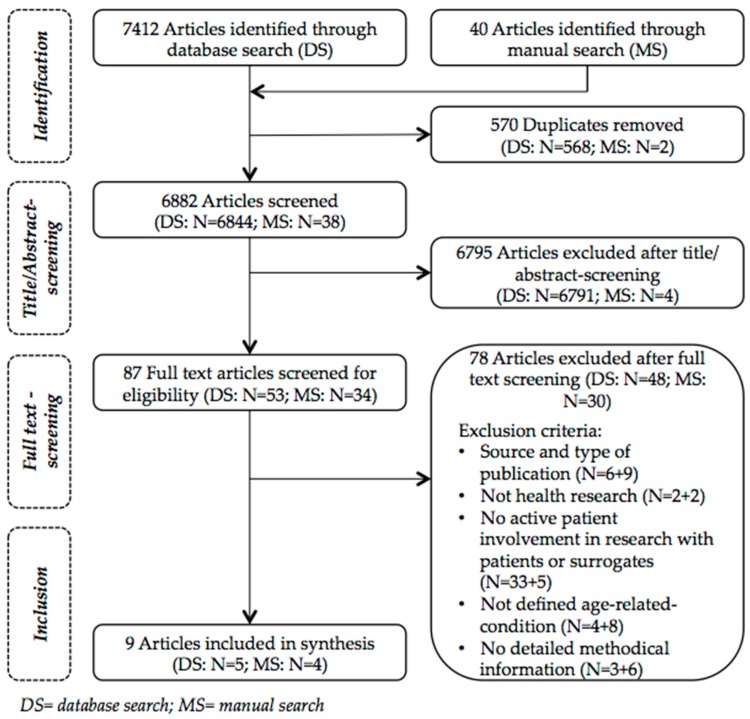
Flowchart for Article Selection.

We used Endnote software (Version X7.7.1, Thomson Reuters) for data management. Databases were searched up to 21 July 2017. After identifying potentially eligible articles (*N* = 7452) and removing duplicates (*N* = 570), one investigator (IS) did a very sensitive screening based on titles and abstracts. Articles were included generously in full text screening if potentially eligible. In total we included 87 articles in full text screening. At the stage of full text screening uncertainties were discussed with the second investigator (AG). We excluded 78 articles after full text screening. For further details on article selection see Figure 2. A list of articles that were excluded at the full-text stage is available from the authors.

### 2.4. Data Extraction and Synthesis

Both authors of this review jointly developed and discussed a standardized form to extract the data. The form was pre-tested on three included articles to ensure usability and completeness of data. IS did the data extraction and AG reviewed the extracted data for correctness. We extracted the following data: reference, topic of article; aim, stages and methods of PPI, participants, recruitment, roles, setting, structure; challenges and facilitators; ethics. All but one item are presented in Table 2 and Table 3. The item ‘ethics’ is described in text only because it was hardly reported. Because the purpose of the review was to get an overview of methods used for involvement, no quality assessment of included articles was conducted. We did a narrative synthesis (as opposed to quantitative synthesis) of the extracted data. We defined our main categories deductively in line with our research questions. Subcategories for ‘challenges’ and ‘facilitators’ were created inductively based on the material. We analyzed common themes across studies.

## 3. Results

A total of nine articles, all conducted in the UK, were included in the review. Eight of them focused on people with dementia [21,24,25,26,27,29,30,31], and one on people with frailty [28]. None of the articles explicitly presented methods for the involvement of people in nursing homes, or with mobility, hearing or visual impairments. Table 2 presents the topics of included articles, as well as the aims, stages and methods of the PPI conducted. Table 3 shows the implementation of PPI in the articles reviewed as well as challenges an involvement.

### 3.1. Implementation of PPI

#### 3.1.1. Aim of Involvement

PPI was done to enhance the connection to the target population [28], the appropriateness of documents [25], the recruitment [21], the interview experiences and data [31] as well as the validity of results [30]. Some methods aimed for improvements beyond the level of single studies: they wanted to enhance the acceptability and feasibility of future research [27] or to improve the use of resources [29] by involving patients or the public.

#### 3.1.2. Methods and Stages of Involvement

The nine articles presented ten studies applying 13 methods of PPI. Iliffe et al. [21] reported on two studies. While most of the methods were used to involve patients and the public in one stage of the research process, one study used PPI in three [31] and two studies in all stages of the research process [26,28]. In the latter the groups involved were referred to as ‘local reference group’ [26] or ‘core reference group’ [28]. Other studies used face-to-face methods such as workshops, focus groups or shared domiciles [21,24,27,29]. One study used ad hoc involvement for flexible one-time consultations additionally to a reference group [28]. Three studies involved the patients’ and public perspectives via survey or postal feedback [21,25,29].

#### 3.1.3. Participants 

Three studies involved only people with own experiences of the old­-age-­related condition [24,30,31], while three other studies additionally involved carers [25,26,27]. In two further studies professionals were also involved [21,29]. A total of eight studies worked only with participants that spoke as private individuals [21,24,25,26,27,30,31], while two worked with both private individuals and organizational representatives of the target population [28,29]. The numbers of PPI participants varied between the studies, ranging from a minimum of three co-researchers in one project [31], to a maximum of 1562 individuals who participated in a survey [29].

#### 3.1.4. Recruitment 

Most recruitment strategies applied in the studies build on the use of disease­-specific support organizations and networks [25] or research centers and networks [27] as distributors. They either shared the requests with all their members (e.g., via newsletters, open offers) [31], or approached selected members (e.g., via group meetings) [26]. Heaven et al. [28] used their core reference group’s personal networks for ad hoc involvement of additional individuals and groups. Two studies built on already existing groups: for the three­-day workshops in a shared domicile all members of the primary study were asked to participate in the PPI [24]. In a project that applied co-research, an existing service user panel from the Alzheimer’s Society (London, UK) was asked to participate [30]. The broadest recruitment strategy applied made use of websites, organizations, social media and local offices to attract participants to their survey [29].

#### 3.1.5. Level of Participation and Roles

Many studies involved participants as consultants who advised on special aspects of the research process [27,28], took part in discussions [21] or gave (anonymous) feedback on the study material [21,25]. Some participants functioned as partners [24,31] and shared control with researchers [26]. The study that used both a reference group and ad hoc involvement stated that these could be used interchangeably for consultation, collaboration and co-production in the PPI process [28].

#### 3.1.6. Setting of Involvement

There were two settings used for PPI activities: either the participants met at a shared venue or they did their PPI task individually. Out of the thirteen methods used for PPI in the studies, four were conducted at venues the participants were familiar with (e.g., within support organizations, regular meeting points of groups, at home) [27,29,30,31]. In one study domiciles were shared with participants for three days at venues the participants were not familiar with [24]. For four methods, the authors did not state any details regarding the setting other than it was a common location [21,26,28,29]. In three methods, survey or postal consultation, the PPI tasks were conducted individually [21,25,29]. For the ad hoc involvement no information on the setting was given [28].

#### 3.1.7. Ethical Approval

Only two of the included studies mentioned obtaining ethical approval [30,31] while three stated that they did not require ethical approval [24,27,29]. Of the latter, one used consent forms and informed participants repeatedly that their participation is voluntary [24]. No information on ethical aspects in the articles was given for the remaining five studies [21,25,26,28].

### 3.2. Practical Challenges and Facilitators for Involvement

For all but two studies the authors stated that they faced various challenges trying to involve people with old-age-related conditions in their studies (see Table 3). The two studies did not report any challenges or facilitators [21,29].

#### 3.2.1. Diversity

The enablement of an effective involvement of diverse people and the avoidance of tokenism was experienced as a challenge [27]. We identified two strategies to deal with diversity used in the included studies: the use of wide networks to ensure diversity in recruitment [28], and the use of separate PPI activities for people with different conditions and experiences [27].

#### 3.2.2. Communication

The authors of the studies included in the review reported various challenges that affect the communication and collaboration for studies with people with dementia. These arose from poor memory, slow cognitive progressing, limited chronological reference, reduced confidence to make a contribution due to participants realizing that their abilities are vanishing and leading to a tendency to agree with researcher’s suggestions [24,30,31]. The studies reviewed used different strategies to enhance communication, such as ensuring accessibility of information, adapting information to the group [26,27], securing knowledge through refreshments and summaries [31], use of meaningful and non­-suggestive task [30], setting a pace that is appropriate for all participants [27] and the use of visualizations [24,27,30,31]. For example, Stevenson et al. [30] elaborate that to avoid having a situation where participants just agreed with researcher’s suggestions “[…] during the interviews, particular attention was given to paraphrasing using the participant’s own words […] and to avoid making suggestions as to what the individual might have meant by a particular response”. This also applies to reflections on the choice of tasks: “Based on this experience, it was considered more meaningful to involve the group in identifying themes rather than verifying the interpretations of the research team” [30].

#### 3.2.3. Location

People with old-age-related conditions may have limited mobility [21], so the choice of the venue where involvement takes place is of great relevance. Some of the studies used a venue for participation with which the participants were already familiar [27,29,30,31]. Bartlett et al. [24] chose to share a domicile with the PPI participants with dementia “[…] to allow time for trusting relationships to be formed and creative energies and collaborations to flow in ordinary and outdoor spaces, rather than on University or other corporate premises”. When searching for a domicile for their workshops, Bartlett et al. [24] looked for an accessible, clearly structured and quiet place that offered enough space for work and leisure activities.

#### 3.2.4. Relationship

For people with dementia, bonding can be difficult [24] and this may result in reduced contributions. Hierarchies between researchers and participants can also contribute towards this effect. To enable meaningful contributions, researchers needed to get to know participants well as individuals and relationships had to be renewed regularly [31]. The location also played a role in this aspect as a relaxed environment where involvement activities can take place unprejudiced [30] or sharing a neutral space with PPI participants for a few days spending both free and working time together were chosen [24]. In two studies, developing a personal relationship to the research topic enabled participants to contribute more deeply [27,31].

#### 3.2.5. Timing

For researchers the timing of PPI is essential as involvement takes time (e.g., for building relationships) [21,31]. Further, potential participants may have temporal constraints [25] or get tired more easily [24]. The articles reviewed described different ways of working with time-related challenges. Two studies adjusted the type and level of involvement to less time consuming methods [21,25], one of them reporting, “[…] to manage time constraints and reduce the potential burden on readers, postal consultations were used” instead of personal encounters [25]. Another study invited the participants into an environment that allowed participation on their own terms and set a schedule that included enough time for both discussions and breaks [24]. Three further studies that emphasized flexibility as being very important introduced it by tailoring processes to the needs of the individuals [31], making attendance flexible [26] and using different PPI methods interchangeably as needed [28].

#### 3.2.6. Continuity

The continuity of participation of people with old-age related conditions may be limited due to the progression of illness and related difficulties in care [26]. The issue has additional importance in longitudinal studies [28]. Two of the studies used the way of recruitment as a strategy to compensate for limitations in continuity: while one study recruited new members via the same support organization former participants were recruited [26], another used PPI groups of sub-studies as a source of its core group [28]. Heaven et al. [28] involved organizations “[…] mindful of the fact that individual representatives may come and go”.

#### 3.2.7. Support for Participants

To support involvement, two studies with face-to-face PPI clarified roles with participants at the beginning [27,30]. One of them additionally trained its reference group members on research methods and useful skills, and handed out a glossary of research terms [27]. Tanner [31] individually supported the co-researchers by giving them sufficient time to share their impressions and feelings and offering them additional support. Stevenson et al. [30] stated the lack of training as a limitation of their PPI processes.

## 4. Discussion

The aim of this review was to support scientists who intend to involve older people in health research by systematically identifying and describing studies that involved people with old-age-related conditions and analyzing the associated facilitators and challenges. We searched three databases and also manually searched the reference lists of the nine articles we identified that met our inclusion criteria, as well as a specialist journal and the INVOLVE library. Further, we used citation tracking in Google scholar to find articles that cited the articles we included. Although we searched for PPI covering six conditions, all but one of the included article were on dementia.

The authors of the included articles stated that patients and the public were involved to provide user perspectives in order to improve their studies. Some articles aimed for empowerment, that is, ensuring that the voices of people who are easily overlooked are heard in the research process [29,31].

The included studies used reference groups, co-production, workshops, residencies, focus groups, surveys and postal feedback to include the perspective of patients and the public as PPI methods [21,24,25,26,27,28,29,30,31]. Thus the methods used to involve people with old-age-related conditions were similar to the range of methods Domecq et al. [1] identified in a systematic review on studies on non-specific populations that applied PPI. The depth of participation varied between consultation, collaboration and co-production. While PPI participants mostly had the role of advisors, long-term reference groups and co-production offered deeper involvement as patients shared control [26] and agenda-setting [28], and were partners to the researchers [24,31].

The involvement of people with old-age-related conditions is feasible but comes with challenges which can be grouped into seven categories: diversity, communication, location, relationship, timing, continuity and support. While some challenges are relevant for PPI regardless of the method applied (e.g., diversity and selection of participants), others are more closely related to particular methods only (e.g., continuity of PPI in longitudinal studies).

### 4.1. Issues on PPI that Are Not Related to Specific Methods

#### 4.1.1. Recruitment, Diversity and Equity

Many of the articles we identified reflected on the diversity and representativeness of PPI participants and discussed potential biases for selection. The included studies primarily used condition-specific support organizations and networks to recruit people with old-age-related conditions for PPI. It is however possible that individuals who are recruited via networks are not the ones in most need of empowerment [32]. Some of the authors were even concerned that only those who identify themselves with the condition in focus would get involved [27,31]. Hence, to recruit people with stigmatizing conditions (e.g., dementia, cognitive impairments) the authors recommended the use of a prudent language and a careful reflection on the ways in which potential participants could be approached [27]. Not only may people in advanced stages of diseases be less adequately represented in studies [30,31], a biased selection can also result from uncertainties regarding effort, task and venue [24], different levels of education and experience with research [25] and underrepresentation of ethnic minorities [26]. While the authors of one study emphasized the special efforts they made to recruit underrepresented groups, such as people with dementia, they unfortunately did not elaborate on the efforts in the article [29].

Selection bias can result in a lack of diversity and selection bias and diversity in PPI are closely related to questions on equity. If only selected groups participate, what does this mean for the groups that are not represented? Are individual opinions represented? How should a legitimate selection of participants look like? Researchers need to reflect whom they want to involve [21]: To what degree do they aim for representativeness? Should their PPI participants come with specific abilities or knowledge? In the process researchers however have to be cautious not to mistake the voice of (one) selected individual(s) as the opinion of all people affected by the condition [31].

#### 4.1.2. Ethics

Ethical issues were rarely discussed in the included articles. In general PPI activities often do not seem to require ethical approval as no research is carried out with the participants [33] who are rather involved as experts based on their own experiences. It is only if PPI participants get in direct contact with study participants that ethic committees need to give their approval as this can be a sensitive activity for both sides [33].

As eight of the nine articles included in our review involved people with dementia, specific ethical needs may arise for the PPI activities with regards to consent and information. Rivett [18] reviewed principles for involving people with dementia as research subjects and related them to their active involvement as co-researchers. She found the process model of consent to be a valuable method to deal with assent and dissent. In contrast to the usual consent procedures, the process model consent is sought and reviewed throughout the whole project [34]. The experiences of Tanner [31] illustrated that using a process model of consent works for PPI activities. She got to know her co-researchers with dementia well at the beginning of the study and monitored and reviewed consent in context and over the duration of the research project. The co-researchers got directly involved with study participants. To ensure their wellbeing Tanner [31] talked to them about their feelings.

To guarantee that the process of involvement follows ethical standards, researchers should be familiar with the individual participant’s signs of well- or ill-being and their strengths and weaknesses, making good relationships a prerequisite. Furthermore, potential participants have to be informed about the PPI project in a way that fits their needs and abilities [18]. None of the included articles described how they dealt with this aspect. There was, however, general information on strategies used to communicate when people with old-age-related conditions were already participating in the PPI, e.g., the use of role-plays and visualization [24,27,30,31].

### 4.2. Challenges and Facilitators within the Used PPI Methods

#### 4.2.1. Continuity and Flexibility in Long-Term Involvement

Both studies that employed PPI over the whole research process reflected on the issues of continuity, recruitment and flexibility [26,28]. For longitudinal studies, continuity of participation can be challenging, especially if people with progressive diseases are involved. To address this limitation, studies ensured that they could recruit new participants without big effort. This they did by identifying potential participants early on through using the PPI groups of sub-studies as a recruitment source, or contacting the same support group that the initial PPI participants came from [26,28]. Flexible PPI models (e.g., flexible attendance in meetings [26] or the use of different PPI methods interchangeably [28]) allowed the adjustment of involvement activities to the individual abilities of the participants and to alterations in the research process.

#### 4.2.2. Communication, Setting and Relationships in Face-To-Face Methods

In studies that used short-term face-to-face-methods it was also important to consider a flexible schedule [24,27,31]. Apart from the reference groups, four studies used face-to-face methods. The said studies involved people with dementia via residencies, workshops or sessions in one or two selected stages of the research process and discussed challenges and facilitators [24,27,30,31]. Common issues that came up for face-to-face involvement were communication, visualization and setting.

People with dementia have specific communicative needs and challenges [24,30,31], hence an inclusive understanding of communication skills is fundamental for their involvement [31]. The communication skills needed by the researchers exceed regular moderation skills [24,27,30,31]. One study made use of trained facilitators who attended the PPI session [30]. No study stated that researchers received training for PPI.

An often-used aid to enhance communication and preserve existing knowledge was visualizations (e.g., the use of pictures, colored cards, films, visual games and charts). These were used to prompt discussions, give structure and focus, and to support continuity [24,27,30,31]. Tanner [31] used multicolored cards to guide the co-researchers in the conduction of interviews. The cards visualized the structure that researchers and co-researchers had jointly developed for the interview process.

The choice of setting is important for PPI. Planning PPI meetings in venues that the participants were familiar and comfortable with allowed for relaxed collaboration and further ensured that the place fitted to the individual requirements [27,30,31]. An unfamiliar setting can lead to rejections as was evident in the study by Bartlett et al. [24]. In the said study some people declined to participate as they were unsure about the venue and worried about potential difficulties. This happened despite the fact that the potential participants knew the researchers.

As described earlier on, good, trustful relationships are central to enhance a meaningful and appropriate face-to-face involvement of people with dementia [24,31]. Investing time to get to know participants and their interests well, and also their strengths and weaknesses added to the comfort and safety of the participants [31]. Based on the assumption that the venue shapes relationships and well-being, Bartlett et al. [24] shared a neutral space with their PPI participants for a few days, spending both, free and working time together. This not only enhanced trust and reduced hierarchies, but also strengthened the group [24].

Participants of PPI may need guidance and support to be able to contribute meaningfully. While there is a discussion in the literature on how far PPI participants need training and how training affects the ‘lay perspective’ participants will bring to research [35], many authors argue that offering training on research skills for the participants can enable them to contribute effectively [2,36]. We found that only one study reported offering training on research methods and useful skills for their participants [27]. The authors of another study rather stated the absence of training as a limitation of their PPI [30].

#### 4.2.3. Individual Tasks as Alternative to Face-to-Face Methods

Three of the ten studies we identified involved the perspectives of patients and members of the public via postal consultation methods [21,25,29]. Two of them discussed their decision against face-to-face methods and for less comprehensive methods [21,25]. Both these studies reported on considerations on timing [21,25], with one of them assigning these to time constraints of potential participants [25]. Difficulties associated with limited mobility and wide spread networks also influenced the decision for individual tasks [21]. Involvement methods with a common setting come with large organizational needs (e.g., to find an accessible venue, organize transport to the destination and assistance, plan a time structure that is feasible for everyone). All this requires an early planning of the PPI [21].

The two studies also reported on the limitations of their choice of postal PPI methods. One of them mentioned the limited depth of information gained through PPI [21]. Burnell et al. [25] indicated that the lack of peer exchange could be a limitation and feared that the goal to empower PPI participants can be missed as patients “may have lacked ownership of the process, perhaps limiting their contributions” [25]. Some of the studies included in the review aimed for empowerment of patients and the public through PPI [29,31]. Postal and other less comprehensive methods risk failing true empowerment of the easily overlooked.

### 4.3. Limitations of This Review

Our review included articles that reported on methods for involving people with at least one of the pre-defined old-age-related conditions in research. As Fudge et al. [32] already reported in a review in 2007, ‘involving people in research’ does not have a common meaning within the research community. We strictly followed INVOLVE’s definition of PPI in research as “research being carried out ‘with’ or ‘by’ members the public rather than ‘to’, ‘about’ or ‘for’ them” [9]. Hence we excluded related approaches such as participatory research whenever it was not designed as research ‘with’ or by members of the public, thereby narrowing our inclusion process. Through this strategy we may have missed certain methods that were employed in studies applying a different definition of involvement.

Almost half of the included articles were not identified through the systematic search of databases but through manual searching. Two of these were found in the new specialist journal ‘Research involvement and engagement’ that is not yet listed in PubMed [27,28]. The other two articles were listed in PubMed but did not yet have keywords [26,30]. Thus, it is possible that we missed relevant articles that were not listed in the databases we searched or were not yet assigned keywords. We limited the search to health-related databases, as we did not expect to identify additional articles through databases from other fields.

The categorization of methods was limited as studies used non-standardized names for processes of involvement. For example, two articles called their PPI ‘co-research’ [30,31] although the depth of involvement was quite different between the two. While one study involved a group of people with dementia in a single two-hours analysis session [30], the other collaboratively prepared, conducted, and analyzed interviews over an extended period [31]. Similarly, a clear assignment of participation levels to methods was not always possible as relevant details, (e.g., the decision-making process), were not sufficiently elaborated in the articles.

We reported the roles PPI participants had in the studies as the authors of the articles described them. As a comprehensive assessment of participants’ experiences was lacking in the articles, we were not able to state how they experienced their roles and the PPI processes. Future research should reflect more on the perspectives of all people involved in PPI processes.

We identified eight articles on PPI with people with dementia, and one on PPI with people with frailty. In contrast, we were not able to identify any article on PPI with people in need of care who live in a nursing home, or people with limitations in hearing, vision or mobility. It is possible that there are articles on the latter conditions that we did not find due to the search terms and selection criteria we used. To ensure that we do not miss relevant articles, we conducted manual searches in specialized journals, reference lists of included articles, and used citation tracking in Google scholar to identify articles citing the articles we included. As has already been mentioned, we excluded articles on participatory action research projects due to the fact that they did not fit with the definition of PPI we used. As a lot of these projects involved people in need of care and living in nursing care homes, we inadvertently simultaneously excluded the only articles that focused on this condition. We do not know in how far the methods, challenges and facilitators identified in the included studies for dementia and for frailty also apply to the other conditions. We can only assume that some aspects are comparable, e.g., the needs for accessible locations, inclusive communication, flexible structures and support. Other aspects might, however, need different approaches: e.g., most of the identified recruitment strategies are based on the use of networks and support organizations which might not be feasible for reaching people living in nursing care homes.

Our aim was to analyze the methods, challenges and facilitators to involve people with old-age-related conditions in research. We found that PPI is feasible but comes with several challenges. As we did not compare the challenges we identified for this group with those of PPI with different user groups (e.g., children, people with cancer), we cannot say whether these challenges are specific for the group in focus or not.

The fact that our review focused on people with old-age-related conditions might give the impression that PPI with people with old-age-related conditions is only a burden. We did not report on the many aspects PPI had a positive impact on in the included studies. Impact occurred at different levels including on the study through a higher quality of the research [26], on researchers through a richer understanding of patients perspectives [30], and on participants through a feeling of empowerment [31].

## 5. Conclusions

In the last years there has been a growing awareness that patients and the public should be more actively involved in the design, conduct and dissemination of health-related studies. In an aging society the need for research on healthcare for people with old-age-related conditions increases. In this context PPI can add to the development of equitable healthcare solutions, democratize research and empower patients who are easily overlooked. Our review adds to the so far limited body of research on how to involve people with old-age-related conditions in research.

Our results demonstrate that PPI with people with old-age-related conditions is feasible. Nonetheless, there can be specific challenges that need to be taken into account. These comprise specific communication needs, limited mobility, temporal constraints, limited continuity of participation, difficulties in bonding, and limited confidence to contribute. We found that both structural and individual aspects need to be considered when aiming for more equity concerning whose perspectives are (effectively) included in research and healthcare. Aspects such as flexibility, accessibility and a respectful attitude that values individual abilities allow a wider range of individuals and groups to get involved in an effective way. Structural considerations on involving diverse patients and members of the public can lead to a more inclusive environment. Furthermore, researchers need to be flexible and plan enough time and resources for the individual needs of the people they involve. Further research is needed on the involvement of people in care homes or with limitations in vision, hearing or mobility. The use of explicit and consented terms within PPI would help to make PPI process more transparent and comparable.

## Figures and Tables

**Table 1:** Eligibility Criteria.

No.	Category	Criteria
**1**	Date of publication	1 January 2007–21 July 2017 (up to 8 August 2017 for manual search)
**2**	Language	English, German, French, Spanish
**3**	Source and type of article	Peer-reviewed journals, empirical articles
**4**	Field of research	Health research
**5**	Active patient involvement in research	Patients or their surrogates are actively involved in the research process as reported by the authors of the article
**6**	Old people	Patients have at least one of the defined old-age-related conditions
**7**	Methods of involvement	Methods of involvement in research are described detailed enough to answer at least one of our research questions

**Table 2:** Overview of references and methods of patient and public involvement (PPI).

References	Topic of Article	Aim, Stage and Method of PPI (as Described by Authors)
Bartlett et al. [24]	Issue of place when involving people with **dementia** in research	**Aim:** Engage participants of primary study in dissemination of research findings **Stage:** Dissemination **Method:** Co-production in shared domicile
Burnell et al. [25]	Involvement of service users in development of intervention study for carers of people with **dementia**	**Aim:** Ensure understandability and appropriateness of information sheets for study participants **Stage:** Development of study information **Method:** Anonymous postal reader consultation (Additional PPI done with family carers to inform intervention for carers)
Giebel et al. [26]	Impact of involvement of people with **dementia** and informal carers in a study program on home care support	**Aim:** Opinions of users shall be considered in research process **Stages:** Proposal; design; data collection (state of PPI at the time of publication) **Method:** Local reference group (Additional PPI done with informal carers in virtual group)
Hassan et al. [27]	Improve research with health devices in **dementia** through PPI	**Aim:** Enhance acceptability and feasibility of future research on health devices in dementia **Stages:** Development of research platform and guide; identification of research questions **Method:** Workshop (Further PPI done with different groups, e.g., researchers, people with mild cognitive impairment, dementia <65 years, no known memory problems)
Heaven et al. [28]	Methods for PPI in a cohort multiple RCT on **frailty** in primary care	**Aim:** Include diverse perspectives in original study and whole program, connect program to target population **Stages:** Proposal; design; data collection; analysis; dissemination **Methods:** (a) Core reference group; (b) Ad hoc groups and individuals
Iliffe et al. [21]	Impact of centrally organized PPI body in three case studies in clinical research (thereof two studies with regard to **dementia**) *Article reports on two studies*	**Aims:** (a) Explore low recruitment rates in on-going study on dementia treatment option; (b) Widening discussion about appropriate ways of introducing topic of dementia with people with Parkinson’s disease **Stages:** Recruitment; Development of study information **Methods:** Centrally organized PPI body offers PPI support to individual studies. Resulting in: (a) PPI focus groups; (b) Individually written feedback
Kelly et al. [29]	Identification and prioritization of unanswered research questions relating to **dementia**	**Aim:** Improve use of resources by integrating stakeholders in priority setting **Stage:** Identification and prioritization of research questions **Methods:** (a) Survey; (b) Prioritization within organizations; (c) Prioritization workshop
Stevenson et al. [30]	Involving co-researchers with **dementia** in analyzing research findings on risk communication in care	**Aim:** Enhance validity in analysis through applying multiple perspectives **Stage:** Analysis **Method:** Co-research (Further PPI done but not elaborated)
Tanner [31]	Involving co-researchers with **dementia** in investigating experiences with transitions between services	**Aim:** Facilitate deeper exchange, good interview experience, richer data **Stages:** Preparation; data collection; analysis **Method:** Co-research

**Table 3:** Implementation, Challenges and Facilitators of PPI.

References	Implementation of PPI	Practical Challenges and Facilitators
Bartlett et al. [24]	**Co-production in shared domicile** Aim(s)/Tasks: Plan dissemination Participants: 7 people with dementia Recruitment: All 16 participants from primary study were invited to participate Setting: Large domicile rented for involvement with both space to be alone and to work together Roles: People with dementia as collaborators in research Structure: Two three days meetings, each in shared domicile, one at beginning and one at end of the dissemination project Participants were accompanied by relative or carer to shared domicile Mixed activities: work (art-based), social (outdoor and indoor activities, conversations) and free time Art used as mediator to generate ideas, enhance communication, results displayed to strengthen continuity, use of visual prompts to focus on task Outdoor activities to strengthen group	**Challenges:** Hierarchies between researchers and people with dementia Reduced confidence to contribute in people with dementia (due to noticing of vanishing abilities) Bonding with new people hard for people with dementia Many people with dementia get tired easily **Facilitators:** Sharing of neutral, barrier-free place: enhances trustful relations, reduces hierarchies in terms of shared power Environment/structure that allow to participate on own terms Thoughtful scheduling: enough time for discussions and breaks Appreciation of contributions and skills of individuals Visual prompts/art: enhances communication, focus, continuity Shared quality time: provides new strength to group Activities outdoor: enhances cognitive/emotional involvement
Burnell et al. [25]	**Anonymous postal reader consultation** Aim(s)/Tasks: Give feedback on understandability and appropriateness of documents via feedback forms Participants: 11 people with dementia and 6 family carers returned feedback (12 in each group were asked to participate) Recruitment: Via two disease- and carer-specific networks Setting: Individual task not bound to location Roles: Service users as anonymous advisors Structure: One-time postal consensus method, anonymous Networks forwarded documents for consultation to interested members with research experience via post, participants returned feedback anonymously to research team by post Feedback forms consisted of scales for rating and open space for individual feedback	**Challenges:** Time constraints of potential participants Participation as burden **Facilitators:** Less time-consuming methods: e.g., one-time postal consultation
Giebel et al. [26]	**Local reference group** Aim(s)/Tasks: Advice on different aspects of study program Participants: 11–15 participants: people with dementia (who have experience with lay involvement), informal carers, members of research team Recruitment: Via group meetings of disease-specific support organization Setting: Meetings take place at one location, reimbursement of travel expenses Roles: People with dementia and carers as advisors and collaborators, shared control with research team members Structure: Biannually face-to-face meetings, cyclic process of involvement Topic selection prior to meeting, meeting with update on previous contributions and input on current topic, integration of feedback in research, meeting notes shared with participants Allowances paid to participants	**Challenges:** Limited continuity of participation due to progression of dementia and associated caring difficulties Reading and speaking difficulties **Facilitators:** Accessible material: plain language, large fonts, additional formats (e.g., audio) Flexible participation: meetings may be missed Recruitment of new PPI members via the same group as initial PPI members: enhances continuity Peer support in local group
Hassan et al. [27]	**Workshop** Aim (s)/Tasks: Discuss (and test) health devices, share experiences, make recommendations for research Participants: 5 people with dementia and 4 carers (age: all but one > 65 years), researchers experienced with PPI and in working with people with dementia Recruitment: Via local dementia resource center and network Setting: Local dementia resource center Roles: People with dementia and carers as advisers Structure: Workshop with two sessions over 1–2 weeks, one week of voluntary device testing in-between: Session 1: Introduction of research field and devices, discussions and testing of devices; Testing: Voluntary device testing at home; Session 2: Discussion of experiences and research suitability of devices, reflection on research requirements Workshop results documented, checked for accuracy with participants, shared with wider research team Guides developed to support sessions and testing (e.g., on devices); research scenarios given for context and to prompt discussion; technical support for device testing Allowances paid to participants	**Challenges:** Effective engagement of diverse people Avoidance of tokenism **Facilitators:** *Workshop design*: Interactive, hands-on experiences Appropriate pace Visual aids Written material adapted to group Discussions in group Home testing of devices as opportunity to contribute individually *Practicalities*: Good environment: setting, time frame, language, instructions Offer of guidance and support Clarification of PPI roles
Heaven et al. [28]	**Core reference group** Aim(s)/Tasks: Monitoring and consultation for original study; overview of whole program; linkage between original and sub-studies; connection to local networks Participants: Organizational representatives from target population (e.g., people over 75 years with frailty), members of research team Recruitment: Via local or research groups Setting: Local meetings (no further information given) Roles: Members of reference group engaged in consultation, collaboration and co-production as appropriate Structure: Quarterly meetings plus interim activity (e.g., facilitation of events, networking) Focus of meetings set by lay members, chaired by project manager as lay representatives declined role Discussions and results documented and fed into the program (reasons for in-action noted) Allowances paid to lay members **Ad hoc groups and individuals** Aim(s)/Tasks: Flexible consultation activity on request, maintain diversity Participants: Groups and individuals from target population (e.g., people over 75 years with frailty) Recruitment: Via networks of core group Setting: Not available (n.a.) Roles: Participants as one-off advisors Structure: Groups and individuals flexibly engaged when needed	**Challenges:** Continuity of involvement in longitudinal studies Wide range of topics in complex studies **Facilitators:** Flexible PPI models in complex studies Involvement of organizational representatives instead of individuals: ensures continuity Own PPI in sub-studies/study sites: reduces workload Groups of the sub-studies used as recruitment resource for core group; people leaving core group can remain involved at less formal levels Training for lay members; glossary of research terms
Iliffe et al. [21]	*Article reports on two studies with different PPI methods:* **(a) Focus groups** Aim(s)/Tasks: Diagnose aspects interfering with recruitment Participants: 27 patients with mild dementia, carers, 2 people without dementia experience Recruitment: Via 2 local specialized research networks Setting: Local (no further information given) Roles: Patients and carers as discussants Structure: One-time involvement, 2 separate focus groups (no further information given) **(b) Individual written feedback** Aim(s)/Tasks: Review patient and carer information sheet for study Participants: 15 external PPI panel members responded to request (consisting of people with experience with dementia as patient or carer, patients with Parkinson’s disease) Recruitment: Via local specialized research networks Setting: Individual, not bound to location Roles: Panel members as individual reviewers Structure: External PPI one-time individual request to external panel members Local research networks shared request and review material with members of own panels; interested members returned their review individually Recommendations and contradictions discussed in original study team with own PPI members (study has two own lay researchers on steering group, not elaborated in article)	**Challenges:** (a) n.a. (b) Authors state that group discussions would be more favorable for task, individual feedback chosen because of: Time considerations Limited mobility of patients in combination with wide spread research networks **Facilitators:** (a) n.a. (b) Group discussions facilitated by: Early planning of PPI in development of study Large organizational force
Kelly et al. [29]	**Survey** Aim(s)/Tasks: Identify unanswered research questions Participants: 1563 individual stakeholders (4% people with dementia, 76% relatives, 15% professionals, others) Recruitment: Via various ways e.g., websites and material of (partner) organisations, social media, local offices; special efforts to recruit underrepresented groups (e.g., people with dementia) (no further information given) Setting: Not bound to location Roles: Stakeholders as individual respondents Structure: One-off survey Survey in online or article form, with open text boxes **Prioritization within organizations** Aim(s)/Tasks: Rank questions for prioritization Participants: 61 organizations representing patients, carers, professionals Recruitment: Via networks Setting: Within organizations Roles: Representing perspective of organization Structure: One-off task Method not set, each organisation chose appropriate method (e.g., consultation, individual decision) and reported process	**Challenges:** n.a. **Facilitators:** n.a.
Kelly et al. [29]	**Prioritization workshop** Aim(s)/Tasks: Reach consensus in prioritization Participants: 18 organizational representatives (among them 2 people with dementia, 5 relatives); Recruitment: Via networks Setting: Capital city Roles: n.a. Structure: One-time workshop Small-group sessions and ranking exercises Documents and task send out in advance Speaking time for each participant, open debate enhanced by facilitator	**Challenges:** n.a. **Facilitators:** n.a.
Stevenson et al. [30]	**Co-research** Aim(s)/Tasks: Identify themes in analysis Participants: 4 people with dementia (age: 2 participants < 65 years, 2 participants 70–79 years), researchers Recruitment: Existing service user research panel from Alzheimer’s Society asked to participate and agreed Setting: Regular venue of Alzheimer’s Society Service User Review Panel Roles: People with dementia as co-researchers, referred to as members of research team in analysis session Structure: Two hours analysis session Attendance of familiar facilitators Presentation of project and clarification of role as co-researcher Interactive exercises: presentation of interview extracts via role play and handout, time for reflection and conversion, prompts to enhance discussion, connection of data to own experiences opened discussion, visualizations as reminder	**Challenges:** Tendency to agree with researcher’s suggestions in people with dementia No training of research skills (*authors state this as limitation*) **Facilitators:** Meaningful, not suggestive task Communication skills: listening, reflecting back in co-researchers own words, positive regard Relaxed and non-judgmental environment Visualization and prompts
Tanner [31]	**Co-research** Aim(s)/Tasks: Preparation of interviews, development of framework, conduction of interviews, first analysis of content and process Participants: Researcher plus 3 people with mild to moderate dementia (age: 60–78 years; gender: 2 males, 1 female; all living at home with their partners) Recruitment: Local dementia service partner published information via various ways (e.g., newsletter, memory café) Setting: Own home and group room for preparation, space of interviews not stated Roles: People with dementia as co-researchers, collaboration Structure: conduction of interviews plus three preparation sessions and two post-interview sessions Preparation sessions: Co-researchers narrated own experiences to enhance their understanding of project and develop interview framework; training of interview skills through reciprocal questions and reflection upon these Prior to interviews: Refreshment of previous meetings Interviews with people with dementia: conducted in partnership (1 researcher, 1 co-researcher); co-researchers did interviews as autonomously as possible; researcher responsible for structure, flexible support of process, technical aspects and quality of research	**Challenges:** Individual challenges in people with dementia Poor memory (information can not be kept) Slow cognitive progressing Limited chronological reference **Facilitators:** Comfortable and familiar venue Enough time to build and renew relationships/trust Knowledge of participants individual strengths and weaknesses: enhances their potential and comfort, ensures quality of research project Use of co-researchers’ own words instead of technical vocabulary Inclusive interpretation of communication skills Refreshing of previous results, use of summaries Visual prompts as memory aids: e.g., for structure
	Immediately after each interview: First analysis of content and process in conversation between researcher and co-researcher, space for co-researchers to talk about own feelings Post-interview sessions: discussions on key themes and issues	Time and money to refresh knowledge and maintain relations Model of process consent: monitor and review consent in context and over duration of project Independent support offered for co-researchers
